# The detrimental effects of recruitment maneuvers on mucus clearance in an animal model of primary ards

**DOI:** 10.1186/2197-425X-3-S1-A308

**Published:** 2015-10-01

**Authors:** G Li Bassi, C Chiurazzi, E Aguilera Xiol, D Marti, C Talitho, R Amaro, C Travierso, M Carbonara, M Rigol, O Comino-Trinidad, A Torres

**Affiliations:** Hospital Clinic de Barcelona, Pulmonary and Critical Care Medicine, Barcelona, Spain; University of Milano. Fondazione IRCCS Ca' Granda Ospedale Maggiore Policlinico, Milan, Italy; Pontifícia Universidade Catolica, CNPq fellow (2012-2014), Rio Grande do Sul, Brazil; University of Barcelona, Barcelona, Spain

## Introduction

The recruitment maneuver (RM) is a transient increase in trans-pulmonary pressure to reopen collapsed alveoli. During mechanical ventilation, mucus could be displaced toward the lungs, driven by the inspiratory flow, via a two-phase gas-liquid flow mechanism [1].

## Objectives

We evaluated, in an animal model of primary acute respiratory distress syndrome (ARDS), the effects of different RMs on the displacement of respiratory secretions, gas exchanges, and respiratory system elastance (Ers).

## Methods

Seven pigs (34 ± 1.8 Kg) were intubated and mechanically ventilated. Animals were challenged into the lungs with *P.aeruginosa* to develop ARDS. After 48 hours from bacterial inoculation, we applied, in a randomized sequence, four different RMs: 1) extended sigh, in volume control (VC) mode, through stepwise increments of 5 cmH_2_O of positive end expiratory pressure (PEEP), every 30 sec, up to 40 cm H_2_0; 2) maximal recruitment strategy, in pressure-control (PC) mode, starting with PEEP of 25 and driving pressure of 15 cmH_2_0, then sequential increments of 5 cmH_2_O of PEEP, every min, up to 35 cmH_2_0; 3) sustained inflation, through continuous positive airway pressure held at 40 cm H_2_O for 30 sec; 4) sudden increase in driving pressure and PEEP in PC-mode, through an increase of PEEP to 16 and driving pressure to 24 cmH_2_O for 90 sec. A 1-hour washout period was allowed between interventions. At baseline, and throughout each RM, mucus transport was assessed through fluoroscopic tracking of radiopaque disks, insufflated into the airways. Mucus clearance velocity (MCV) was computed. Positive and negative MCV values describe mucus moving toward the glottis and lungs, respectively. After 15 min from completion of each RM, arterial partial pressures of oxygen/inspiratory fraction of oxygen and carbon dioxide (Pa02/F_I_02 and PaC02), and Ers were assessed and adjusted per baseline values.

## Results

At baseline, animals were ventilated with respiratory rate of 40 ± 10 breaths/min, tidal volume 260 ± 14 mL, and PEEP 8.5 ± 0.5 cm H_2_O. As a result, Pa02/F_I_02 and PaC02 were 277 ± 100 and 51 ± 13 mmHg, respectively. The effects of RMs on MCV, Ers, Pa02/F_I_02 and PaC02 are reported in table 1. MCV, during RMs that either improved or impaired Pa02/F_I_02, was 0.0 ± 0.5 and -0.2 ± 1.0 mm/min, respectively (p = 0.591).

**Table 1:** Table 1

	Baseline	Extended sigh	Maximal recruitment strategy	Sustained inflation	Sudden increase driving pressure and PEEP	P-value
Mucus Clearance (mm/min)	2.2 ± 2.4	0.3 ± 0.9	-0.2 ± 0.9	-0.3 ± 0.6	-0.1 ± 0.7	0.021
Incidence of mucus moving toward lungs (%)	0	50.0	57.1	80.0	57.1	0.013
Respiratory System Elastance After-Before recruitment maneuver (cm H20/L)	NA	-5.8 ± 3.5	-9.0 ± 8.9	-4.1 ± 3.1	-3.6 ± 3.9	0.124
Pa02/FI02 After-Before recruitment maneuver (mmHg)	NA	4.1 ± 63.1	2.8 ± 45.8	13.6 ± 42.3	-2.2 ± 41.6	0.937
PaC02 After-Before recruitment maneuver (mmHg)	NA	6.9 ± 7.8	3.9 ± 7.4	6.7 ± 13.6	1.8 ± 4.0	0.616

*[Effects of recruitment maneuvers]*

## Conclusions

In a model of primary ARDS, we found that different methods to recruit the lungs produce similar results on gas exchanges and Ers, but consistently impair mucus clearance. Likely, the distinctive inspiratory airflow patterns generated by RMs are major culprit in these findings.

## Grant Acknowledgment

2013 ESICM/ECCRN young investigator award.
